# Genomic Analysis of Carbapenemase-Producing Extensively Drug-Resistant *Klebsiella pneumoniae* Isolates Reveals the Horizontal Spread of p18-43_01 Plasmid Encoding *bla*_NDM-1_ in South Africa

**DOI:** 10.3390/microorganisms8010137

**Published:** 2020-01-17

**Authors:** Yogandree Ramsamy, Koleka P. Mlisana, Mushal Allam, Daniel G. Amoako, Akebe L. K. Abia, Arshad Ismail, Ravesh Singh, Theroshnie Kisten, Khine Swe Swe Han, David J. Jackson Muckart, Timothy Hardcastle, Moosa Suleman, Sabiha Y. Essack

**Affiliations:** 1 Antimicrobial Research Unit, College of Health Sciences, University of KwaZulu-Natal, Durban 4000, South Africa; lutherkinga@yahoo.fr (A.L.K.A.); essacks@ukzn.ac.za (S.Y.E.); 2 Medical Microbiology, National Health Laboratory Services, Durban 4000, South Africa; MlisanaK@ukzn.ac.za (K.P.M.); Singhra@ukzn.ac.za (R.S.);; 3 Medical Microbiology, College of Health Sciences, University of KwaZulu-Natal, Durban 4000, South Africa; 4 Sequencing Core Facility, National Institute for Communicable Diseases, National Health Laboratory Service, Johannesburg 2131, South Africa; MushalA@nicd.ac.za (M.A.); ArshadI@nicd.ac.za (A.I.); 5 School of Clinical Medicine, Discipline of Anaesthetics & Critical Care, College of Health Sciences, University of KwaZulu-Natal, Durban 4000, South Africa; toshiekis@gmail.com; 6 Inkosi Albert Luthuli Central Hospital, Department of Critical Care, College of Health Sciences, University of KwaZulu-Natal, Durban 4000, South Africa; 7 Inkosi Albert Luthuli Central Hospital, Department of Surgery & Trauma Unit, College of Health Sciences, University of KwaZulu-Natal, Durban 4000, South Africa; davidmuckart@gmail.com (D.J.J.M.); timothyhar@ialch.co.za (T.H.); 8 Ahmed Al-Kadi Private Hospital, Durban 4000, South Africa; msulemanmd@gmail.com

**Keywords:** genomics, carbapenemase, *Klebsiella pneumoniae*, extensively drug-resistant, mobile genetic elements, epidemiology, phylogenomic, South Africa

## Abstract

Whole-genome sequence (WGS) analyses were employed to investigate the genomic epidemiology of extensively drug-resistant *Klebsiella pneumoniae* strains, focusing on the carbapenem resistance-encoding determinants, mobile genetic support, clonal and epidemiological relationships. A total of ten isolates were obtained from patients admitted to the intensive care unit (ICU) in a public hospital in South Africa. Five isolates were from rectal swabs of colonized patients and five from blood cultures of patients with invasive carbapenem-resistant infections. Following microbial identification and antibiotic susceptibility tests, the isolates were subjected to WGS on the Illumina MiSeq platform. All the isolates showed genotypic resistance to tested β-lactams (NDM-1, OXA-1, CTX-M-15, TEM-1B, SHV-1) and other antibiotics. All but one isolate belonged to the ST152 with a novel sequence type, ST3136, differing by a single-locus variant. The isolates had the same plasmid multilocus sequence type (IncF[K12:A-:B36]) and capsular serotype (*KL149*), supporting the epidemiological linkage between the clones. Resistance to carbapenems in the 10 isolates was conferred by the *bla*_NDM-1_ mediated by the acquisition of multi-replicon [ColRNAI, IncFIB(pB171), Col440I, IncFII, IncFIB(K) and IncFII(Yp)] p18-43_01 plasmid. These findings suggest that the acquisition of *bla*_NDM-1_-bearing plasmid structure (p18-43_01), horizontal transfer and clonal dissemination facilitate the spread of carbapenemases in South Africa. This emphasizes the importance of targeted infection control measures to prevent dissemination.

## 1. Introduction

The last decade has witnessed a dramatic increase both in the proportion and absolute number of multi-drug resistant bacterial pathogens [1]. Infections caused by extensively drug-resistant (XDR) Gram-negative pathogens have emerged as one of the world’s greatest threats [2], not the least of which are the carbapenem-resistant bacteria that are on the rise globally [3,4,5]. There is a continuous rise in bacterial resistance, and unfortunately, there are potentially no new drugs on the horizon to replace the existing antibiotics against which resistance has developed [6,7]. This necessitates the urgent search and development of potential candidates in the drug pipeline to help manage this global threat of antibiotic resistance [8,9,10,11].

Carbapenems are a potent class of β-lactam antibiotics that are often used as “last-line agents” or “antibiotics of last resort” when infected patients become severely ill or are suspected of harboring resistant bacteria [12]. They are considered first-line agents in the treatment of infections caused by extended-spectrum β-lactamase (ESBL)-producing organisms [13]. The widespread use of carbapenems for empiric and directed treatment of severe infections has resulted in the emergence of carbapenem-hydrolyzing β-lactamases, also known as carbapenemases, as the most well-recognized mechanism of resistance to carbapenems. These enzymes inactivate all known β-lactams and represent the most versatile family of β-lactamases, with a breadth of spectrum unrivaled by other β-lactam-hydrolyzing enzymes [14]. The production of carbapenemases by *Enterobacterales* such a *K. pneumoniae* results in limited treatment options with an inevitable high mortality rate caused by carbapenemase-producing *Enterobacterales* (CPE) [15,16]. Infection with carbapenem-resistant Enterobacterales has emerged as an important problem that threatens the health and wellbeing of patients in health-care settings [17,18]. The alarming global spread of CPE isolates has reached African countries including in Angola, Algeria, Gabon, Mali, Nigeria, and South Africa with NDM-1 and OXA-48 been the most commonly reported carbapenemases [19,20,21,22,23,24]. This rapid dissemination of CPE is supported by intra- and interspecies plasmid-mediated transfer of carbapenemase-encoding genes detected on a diversity of plasmid backbones [25,26]. Plasmids of several incompatibility groups (Inc) can mediate the spread of carbapenem resistance, mostly resulting in XDR *K. pneumoniae* [27,28]. Till date, a total of 760 *K. pneumoniae* plasmid annotation reports are available at the Pathosystems Resource Integration Center (PATRIC) database (https://www.patricbrc.org/).

Fecal carriage of CPE isolates has been investigated rarely compared with carriage of isolates producing ESBLs [29], particularly in a “non-outbreak” setting. Rapid identification of patients colonized with CPE is an integral part of intervention strategies and infection control measures required to contain hospital infections due to CPE [30]. There is a growing body of evidence that suggests that early detection of patients colonized with CPE on admission to health-care facilities may assist in the prevention of outbreaks limiting the regional spread of this emerging threat [6]. Additionally, understanding the molecular mechanisms of resistance could provide valuable insights into the management of drug-resistant in *K. pneumoniae* infections. Herein, whole-genome sequence (WGS) analysis was employed to investigate the molecular epidemiology of ten XDR *K. pneumoniae* strains (from both colonized and infected patients), focusing on the carbapenem resistance-encoding determinants, their mobile genetic support, clonal and epidemiological relationships in a public hospital in KwaZulu-Natal, South Africa. 

## 2. Materials and Methods 

### 2.1. Ethical Approval

Ethical clearance was granted by the Biomedical Research Ethics Committee of the University of KwaZulu-Natal (approval no: **BE: 453/15,** approval date: 29 February 2016).

### 2.2. Study Site and Sample Collection 

This prospective study was performed at the Inkosi Albert Luthuli Central Hospital (IALCH). IALCH is a centralized healthcare facility located in Durban, KwaZulu-Natal, South Africa. This Level 4 hospital has a Medical and Surgical ICU comprising of 6 beds each. The ten-bed trauma ICU is exclusively for trauma patients who are admitted either directly from an injury scene or transferred from another district hospital within 24 h of sustained injuries unless otherwise specified. Rectal swab specimens to identify CPE were obtained from patients admitted to the Medical, Surgical and Trauma ICU between May 2016 and May 2017. A total of 263 patients were screened. Additionally, clinical isolates of CR *K. pneumoniae* implicated in bloodstream infections were obtained from the same wards in the same period.

### 2.3. Isolation and Identification of Carbapenemase-Producing Klebsiella Pneumoniae Isolates

#### 2.3.1. Culture Screening Methods

Rectal swabs were obtained using a nylon flocked swab system with 5 mL of Amies gel transport medium. The swabs were immediately streaked on ChromID CARBA SMART chromogenic agar medium (BioMérieux, Marcy l’Étoile, France) containing antibiotics that enable selective isolation of and identification of carbapenemase-producing *Enterobacterales*. This media provides rapid and reliable identification of all CPE/CRE, particularly KPC, NDM-1, and OXA-48—producing isolates [31]. Inoculated plates were incubated for 18 to 24 h at 37 °C in ambient air. All ChromID CARBA SMART (BioMérieux, Marcy l’Étoile, France) agar plates were inoculated with the following control strains: carbapenemase-negative *K. pneumoniae* ATCC 700603, and carbapenemase-positive *K. pneumoniae* ATCC BAA-1705.

#### 2.3.2. Detection and Identification of CPE Colonies

Presumptive CPE colonies from isolated from the ChromID CARBA SMART agar were sub-cultured onto MacConkey plates, and pure colonies were phenotypically identified using the VITEK II system (BioMérieux, Marcy l’Étoile, France). Confirmed CRE were then subjected to the RAPIDEC^®^ CARBA NP (BioMérieux, Marcy l’Étoile, France) test to detect carbapenem hydrolysis by carbapenemase-producing bacteria.

### 2.4. Antibiotic Susceptibility Testing (AST)

Antibiotic susceptibility testing was performed, and the minimum inhibitory concentrations (MICs) were ascertained using the VITEK II (BioMérieux Marcy l’Étoile, France) platform. The results were interpreted according to the Clinical Laboratory Standards Institute (CLSI) guidelines [32]. The VITEK II AST-N255 card was used to perform antibiotic susceptibility testing. The universal antibiotic test panel included: penicillin, ampicillin, amoxicillin-clavulanate, ceftriaxone, cefepime, cefuroxime, cefoxitin, ceftazidime, imipenem, meropenem, ertapenem, piperacillin-tazobactam, amikacin, gentamicin, nitrofurantoin, trimethoprim/sulfamethoxazole, ciprofloxacin, and tigecycline. Colistin susceptibility testing was not performed as currently all available laboratory methods are unreliable and may not predict clinical outcome [33]. Isolates were characterized as susceptible or resistant using CLSI breakpoints [32].

### 2.5. DNA Extraction Genome Sequencing and Analysis

The isolates were grown on nutrient agar (Oxoid, UK) and incubated overnight at 37 °C prior to genomic DNA extraction. Genomic DNA (gDNA) was extracted using the GenElute^®^ bacterial genomic DNA kit (Sigma–Aldrich, St. Louis, MO, USA) according to the manufacturer’s instructions. The quantification of extracted gDNA was determined on a Nanodrop spectrophotometer, Qubit, and verified on an agarose gel electrophoresis. Multiplexed paired-end libraries (2 × 300 bp) were prepared using the Nextera XT DNA sample preparation kit (Illumina, San Diego, CA, USA) and sequences were determined on an Illumina MiSeq platform with 100× coverage at the National Institute of Communicable Diseases Sequencing Core Facility, South Africa. The resulting raw reads were checked for quality, trimmed, and *de novo* assembled into contigs using the CLC Genomics Workbench version 10 (CLC, Bio-QIAGEN, Aarhus, Denmark) [34]. The *de novo* assembled reads were uploaded in GenBank and annotated using NCBI prokaryotic genome annotation pipeline and RAST 2.0 server [35], which identified encoding proteins, rRNA and tRNA, assigned functions to the genes and predicted subsystems represented in the genome.

### 2.6. WGS-Based Confirmation and Molecular Typing of K. Pneumoniae Isolates

The generated contigs from the WGS data were used to confirm the *Klebsiella pnuemoniae* isolates using the SpeciesFinder 2.0 platform (https://cge.cbs.dtu.dk/services/SpeciesFinder/) which predicts the genus and species the strains in-silico. Multilocus sequence typing (MLST) was performed in-silico using the WGS data online platform tool from the assembled genomes (https://bigsdb.pasteur.fr/klebsiella/klebsiella.html) which also predicted the allelic profiles of the seven housekeeping genes, *gapA*, *infB*, *mdh, pgi*, *phoE*, *rpoB*, and *tonB* of *K. pneumoniae*. The reference *Klebsiella* WGS data online platform tool, Kaptive-web (http://kaptive.holtlab.net/) was used to infer the serotypes (*K* types, *wzc* and *wzi* allelic types) of the isolates.

### 2.7. WGS Identification of the Acquired and Chromosomal Mutations in the Isolates

The bacterial analysis pipeline GoSeqIt (https://www.goseqit.com/web-services/) via ResFinder [36] and the comprehensive antibiotic resistance database (CARD; https://card.mcmaster.ca) [37] were used to annotate and identify antibiotic resistance genes. To detect the molecular basis of resistance (developing by chromosomal SNPs) against quinolones (*gyrA* and *parC*), the nucleotide allele sequences were translated with tBLASTn to call SNPs in these genes. The fluoroquinolone susceptible *K. pneumoniae* ATCC 13883 (PRJNA244567) was used as the reference/wild-type strain. The detected mutations were confirmed using the CARD platform which can equally predict chromosomal mutations.

### 2.8. WGS Identification of Mobile Genetic Elements (MGEs)/Genetic Support

Plasmid replicons were predicted through PlasmidFinder [38]. PHAge Search Tool (PHAST) server was used for the identification, annotation, and visualization of prophage sequences [39]. Insertion sequences (IS) resident in genomes were predicted by uploading contigs on the ISFinder database (https://www-is.biotoul.fr/) [40]. The carbapenemase genes and their flanking sequences obtained from the RAST SEED viewer were searched on the NCBI microbial nucleotide BLAST. Fully sequenced plasmids, with the closet synteny obtained from the BLAST search, were used as a reference input to GView Server (https://server.gview.ca/), together with the 10 annotated Illumina sequence reads of the XDR-*Klebsiella pneumoniae* isolates to visualize the presence/absence of specific plasmid DNA.

### 2.9. Phylogenomic Analyses of the K. Pneumoniae Isolates (n = 10)

The *de-novo* assembled contigs were submitted to CSI Phylogeny-1.4 (https://cge.cbs.dtu.dk/services/CSIPhylogeny-1.2), an online service which identifies SNPs from WGS data, filters and validates the SNP positions, and then infers phylogeny based on concatenated SNP profiles [41]. The genome of *K. quasi-pneumoniae* strain P27-02 (accession number: NXHG00000000.1) served as the outgroup to root the tree enabling the easy configuration of the phylogenetic distance between the strains on the branches. The pipeline was run with default parameters: a minimal depth at SNP positions of 10 reads, a minimal relative depth at SNP positions of 10%, a minimal distance between SNPs of 10 bp, a minimal *Z-score* of 1.96, a minimal SNP quality of 30 and a minimal read mapping quality of 25. The obtained phylogenomic tree was downloaded in Newick format, annotated and visualized or edited using an interactive tree of life (ITOL) (https://itol.embl.de/).

Additionally, a genome-wide gene-by-gene comparison approach was used to assess the phylogenetic relatedness between isolates using Rapid large-scale prokaryote pangenome analysis (Roary; https://sanger-pathogens.github.io/Roary/) to estimate the tree for the core genome. The annotated genome assemblies were used to determine the core genes and predicted coding regions were extracted and converted into protein sequences. A total of 4605 core genes were extracted with an alignment length of 4,294,572 bp shared by the ten *K. pneumoniae* genomes.

The allelic distance from the cgMLST was edited and visualized using Figtree v1.4.3 (https://tree.bio.ed.ac.uk/sofware/figtree/) in a maximum likelihood phylogenetic tree using optimized parameters: nucleotide substitution model, Jukes-Cantor; transition/transversion ratio, 2; estimate substitution rate, yes; number of substitution rate, 4; perform bootstrap analysis, yes; replicates, 1000. The phylogeny was visualized with annotations for isolate demographics, WGS in-silico typing (ST, *K type*), β-lactamases, and mobile genetic elements metadata using Phandango [42] to provide a comprehensive analysis of the generated phylogenomic tree.

### 2.10. Accession Numbers

The raw read sequences and the assembled whole-genome contigs have been deposited in GenBank. The data is available under project number PRJNA411997.

## 3. Results

### 3.1. Identification, Confirmation and Phenotypic Analysis

Five out of the 263 rectal swabs (colonization rate of 1.9%) as well as the five blood culture samples obtained from infected patients for comparison, were confirmed as carbapenem-resistant K. pneumoniae (CRKP). Antibiotic susceptibility testing (AST) revealed that all the isolates were extensively drug-resistant (XDR) (Table 1). The relevant patient data, source of the specimen, and relevant phenotypic features (AST and Carba NP test) for the ten collected CRKP isolates are summarized in Table 1 and Table 2.

### 3.2. Genomic Confirmation and Resistance Profiling of β-Lactamases

The SpeciesFinder platform confirmed all the with generated genomic data as *K. pneumoniae*. The genomic attributes of the 10 sequenced CRKP isolates are shown in Table S1. Resistance to antibiotics was attributed to multiple genes mediating resistance to different antibiotic classes (Table 2 and Table S2).

### 3.3. WGS-Based Capsular Serotyping and Multilocus Sequence Typing (MLST)

The epidemiological typing scheme via the Kaptive database predicted the same capsular polysaccharide serotype [KL149-*wzc*:928, *wzi*:110] for all the isolates. Further MLST-analyses revealed that the 9 of the CRKP strains belonged to ST152 (same clonal lineage) with the allelic profiles (*gapE-2*, *infB-3*, *mdh-2*, *pgi-1*, *phoE-1*, *rpoB-4*, *tnoB-56*) except for the novel ST3136 (*n* = 1) [45] which differed by a single-locus variant (SLV) in the *rpob* allelic gene_85 (Table 2 and Table S2).

### 3.4. WGS Detection of Carbapenemase-Encoding Bla_NDM-1_ Plasmid Involved in Horizontal Spread

**Figure 1:**
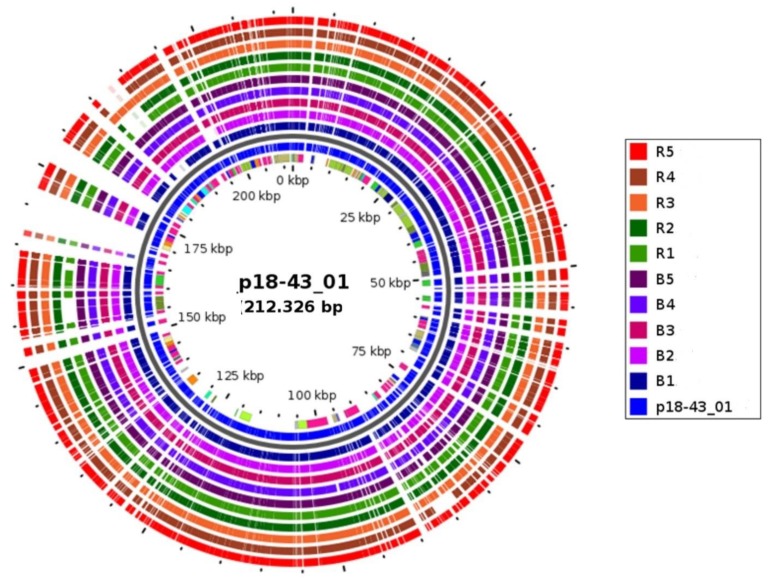
Tracking of plasmid p18-43_01 in NDM-1-encoding CP-*K. pneumoniae* isolates (*n* = 10). The map was constructed using the GView online server (https://server.gview.ca/). The concentric circles represent comparisons between p18-43_01 and, starting with the inner circle, genome assemblies from *Klebsiella pneumoniae* species (strain ID: B1, B2, B3, B4, B5, R1, R2, R3, R4, and R5). Color codes are given for each strain with a plasmid synteny identity, ranging from 99–100%.

All the *bla*_NDM-1_ genes always occurred with bleomycin resistance determinants (ble_MBL_). The NCBI microbial nucleotide BLAST search of the carbapenemase (NDM-1) and its flanking sequences in all the isolates revealed that the *bla*_NDM-1_ was located on a 212.3 Kbp multi-replicon plasmid (p18-43_01; accession no. CP023554.1) (Table 2). Comparative analyses via the GView server (Figure 1) tracked and confirmed the presence of similar DNA synteny with 99–100% coverage and identity to the p18-43_01 reference in all the *bla*_NDM-1_ positive CRKP isolates (Table 2 and Table 3).

WGS analysis via the PlasmidFinder online platform revealed different plasmid replicon types (Inc FIB(K), Inc FII, Inc FIB (pB171), Inc FII(Yp), ColRNAI, and Col440I) grouped into two different combinations in the CRKP isolates (Table 3). The ISFinder predicted 14 insertion sequences in 5 varied permutations (Figure S1 and Table 3).

### 3.5. Phylogenomic Insights

**Figure 2:**
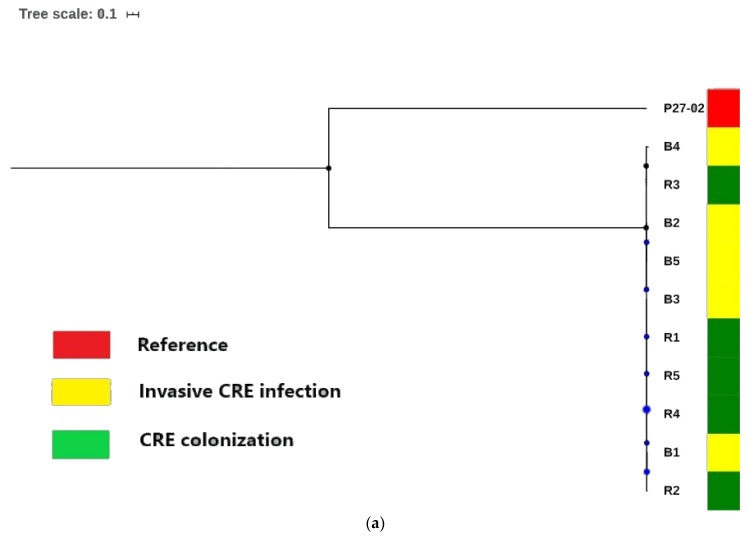

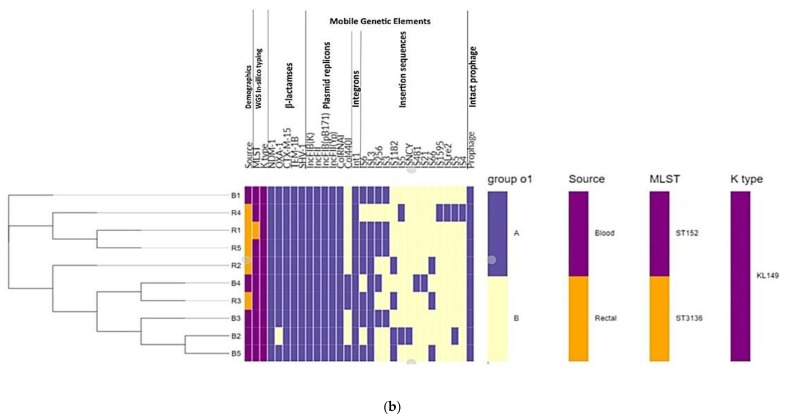
(**a**): A phylogenomic tree based on the single nucleotide polymorphism (SNPs) differences from whole genomes of the 10 carbapenem resistant-K. pneumoniae isolates. The K. quasi-pneumoniae strain P27-02 (accession number: NXHG00000000.1) was rooted and used as the outgroup in the tree. The bootstrap values (in blue dots) for the nodes have been indicated on the tree. The tree depicted a major clade with the node showing the slight differentiation of the isolates in the phylogenetic tree. The scale bar represents one nucleotide substitution per 1000 sequence positions. (**b**): The core genome phylogenetic branch and metadata (demographics; WGS in-silico typing; β-lactamases, plasmid replicons, integrons, insertion sequences, and intact prophages) coupled by the use of Phandango (https://github.com/jameshadfield/phandango/wiki) in isolated carbapenem resistant-*K. pneumoniae* strains (*n* = 10) from the public hospital in South Africa. The color codes for β-lactamases, plasmid replicons, integrons, insertion sequences and intact prophages (10) showed presence (light blue; A) and absence (yellow; B) in the isolates.

Phylogenomic tree analysis based on the single nucleotide polymorphism (SNPs) differences from whole genomes grouped the isolates into a single clade confirming the high genetic similarity depicted in their epidemiological profiles (capsular serotypes and sequence types) (Figure 2a). The tree depicted a major clade with the nodes (bootstrap in blue circular dots) showing a slight differentiation between the CRKP isolates from both colonized and infected patients on the phylogenomic branch (Figure 2a).

Core genome phylogenetics via Roary coupled with metadata analysis, however, provided useful insights into the slight distinctions between the CRKP isolates (Figure 2b). Specifically, there were differences in the plasmid replicons and insertion sequences in the genomes that were possibly associated with variations in the common genetic backbone of the *bla*_NDM-1._

## 4. Discussion

The global dissemination of carbapenemase-producing *Enterobacterales* (CPE) poses a serious threat to public health and clinical practice as these bacteria are resistant to the last-resort antibiotics (carbapenems) and cause high mortality [46,47,48]. The rapid emergence and widespread dissemination of XDR *K. pneumoniae* over recent years are of great concern [49]. As eluded by Yang et al., 2011 antibiotic resistance mediated by plasmids has been increasing at a remarkable rate, especially through genes encoding carbapenemases [50]. Therefore, a thorough understanding of their resistance mechanisms and spread will offer valuable insights into their management. Herein, whole-genome sequence (WGS) analyses were employed to investigate the molecular epidemiology of carbapenem-resistant *K. pneumoniae* strains, focusing on the carbapenem resistance-encoding determinants, mobile genetic support, clonal and epidemiological relationships.

The CRE colonization (or carriage) rate of 1.9% obtained, which was much higher than the 4.2% faecal colonisation with CRKP isolates in a paediatric hospital in South Africa [51]. However, the finding was comparable to a study in the tertiary hospital in Korea by Kang et al. [52], where a CRE carriage rate on admission in 833 adults was 2.8%. Similarly, less than 2% CRKP was recorded in 7- year surveillance study in a primary health care centre in China [53]. However, in Brazil, a 6.8% CRE colonization rate has been reported on admission [54]. The different rates across different settings are not peculiar to the carbapenem-resistant *K*. *pneumoniae* isolates were extensively drug-resistant (XDR) and harbored the New Delhi Metallo-β-lactamase (*bla*_NDM-1_) that mediates resistance to carbapenems (meropenem and imipenem) [55,56]. The detection of *bla*_NDM-1_ carbapenemases in both infected and colonized patients has been reported in South Africa [51,57,58,59], Africa [19,21,56,60] and globally [61]. Furthermore, all the isolates possessed chromosomal mutations, plasmid-mediated quinolone resistance genes, and efflux genes, whose combined effect mediates high-level quinolone resistance [62,63,64].

In-silico *Klebsiella* typing scheme that represents useful epidemiological markers for *Klebsiella* strain serotyping predicted the same capsular serotypes (KL149-*wzc*:928, *wzi*:110). This finding suggests a possible epidemiological linkage between the isolates [65]. Interestingly, the KL149 serotype has been linked with ESBL producing and carbapenemase positive invasive *K. pneumoniae* isolates from South and Southeast Asia (Hong Kong, India, and Vietnam) [66]. Furthermore, MLST analyses revealed the same clone (ST152) for the CRKP isolates except for one which belonged to the novel ST3136 [45] and differed by a single allelic gene affirming the high epidemiological linkage in the isolates and a possible clonal expansion of ST152. While there were no studies on *K. pneumoniae* capsular serotypes in the country for comparison, a study by Agyapong et al. [63] on *K. pneumoniae* isolates from Ghana showed a 100% concordance between 2 typing schemes and reported that ST152 isolates contained a similar capsular serotype to that shown in this study. The slight differences in the two typing results indicate that the MLST is more resolute than capsular polysaccharide serotyping.

Analysis of the genetic backbone of the carbapenemase and its flanking sequences linked *bla*_NDM-1_ to a mobile element p18-43_01 (multi-replicon plasmid) [58] in all CRKP isolates (Figure 1, Table 2 and Table 3). This p18-43_01 plasmid has been reported for the spread of *bla*_NDM-1_ in CRE (including *K. pneumoniae, K. michiganensis, Serratia marcescens, Citrobacter freundii*, *and Enterobacter* spp*.)* in South Africa [58]. This implicates *bla*_NDM-1_ acquisition as well as nosocomial spread and development of an XDR genetic lineage in different species. This is of concern in terms of our last-resort antibiotic arsenals for the treatment of drug-resistant bacteria in the country.

The different combinations of genetic support such as the varied plasmid replicons (Inc FIB(K), Inc FII, Inc FIB(pB171), Inc FII(Yp), ColRNAI, and Col440I) and insertion sequences support the assertion of the versatility in this *bla*_NDM-1_ encoding plasmid backbone structure enabling a local horizontal transfer between isolates (Figure S1 and Table 3). Wenzi Bi. et al., in 2017, reported the dissemination and epidemicity of clinical XDR *K. pneumoniae* strains result from horizontal transmission of multiple resistance determinants via IncF plasmids [67]. More so, the multi replicon nature of the p18-43_01 plasmid was not peculiar, as a diversity of plasmid incompatibility groups (Inc), including IncX, IncR, IncN, IncL/M, IncA/C, and IncF have been linked with NDM variants [26,27,68,69]. The intra- and inter-clonal spread of the *bla*_NDM-1_ plasmid-bearing structure in both ST152 and ST3136 support the findings that NDM-positive *K. pneumoniae* strains of African origin have been multi-clonal [60]. Further insight into the host adaptation and evolution of the p18-43_01 plasmid in the CRKP isolates would require DNA sequence circularization.

Comparative phylogenomic analysis of the 10 CRKP isolates with WGS SNPs analysis corroborated their close epidemiological profiles (capsular serotypes and sequence types), which showed less genetic variation in isolates recovered from the colonized and infected patients (Figure 2a). This has been reported in many species, including *Acinetobacter baumannii* [70]. Furthermore, core genome phylogenetics, combined with metadata analysis, provided useful insights into the slight distinctions (replicons and insertion sequences) between the CRKP isolates (Figure 2b) [71]. This is possibly associated with variations in the common genetic backbone of the *bla*_NDM-1,_ supporting its versatility via a local horizontal transfer and subsequent evolution in their host by recombination events [72,73]. This reiterates the need for further circularization and annotation of the plasmid DNA using sequencing techniques that provide long-read sequences to offer insights into its evolution and spread [74]. Moreover, further larger epidemiological studies should be conducted in the province to trace the primary source(s) of their spread, possibly through frequent contact with healthcare workers and the movement of colonized patients among different healthcare settings [75,76].

## 5. Conclusions

The acquisition of resistance-encoding plasmids, horizontal transfer and clonal dissemination facilitate the spread of carbapenemases in KZN, South Africa, which is very worrisome for infectious disease management and highlights the importance of early detection of CRE and targeted infection control measures to prevent dissemination. Further studies would elucidate the extent of CPE dissemination in this region and identify the primary source(s) of their spread. Such knowledge will enable the development of effective countermeasures against the spread of CPE.

## Figures and Tables

**Table 1:** Antibiotic susceptibility of the *Klebsiella pneumoniae*.

Bacterial Isolate *			MIC (mg/L) ^†^																	
No.	Strain ID	Category	IMP	MEM	FEP	CXM	CTX	CAZ	CRO	FOX	AMP	AMC	TZP	AMX	GEN	AMK	CIP	ERT	SXT	TGC
1	B1	XDR	≥16	≥16	≥64	≥64	≥64	≥64	≥64	≥64	≥32	≥32	≥128	≥32	≥16	≥64	≥4	≥8	≥320	≤0.5
2	B2	XDR	≥16	≥16	≥64	≥64	≥64	≥64	≥64	≥64	≥32	≥32	≥128	≥32	≥16	≥64	≥4	≥8	≥320	2
3	B3	XDR	≥16	≥16	≥64	≥64	≥64	≥64	≥64	≥64	≥32	≥32	≥128	≥32	≥16	≥64	≥4	≥8	≥320	1
4	B4	XDR	≥16	≥16	≥64	≥64	≥64	≥64	≥64	≥64	≥32	≥32	≥128	≥32	≥16	≥64	≥4	≥8	≥320	2
5	B5	XDR	≥16	≥16	≥64	≥64	≥64	≥64	≥64	≥64	≥32	≥32	≥128	≥32	≥16	≥64	≥4	≥8	≥320	2
6	R1	XDR	≥16	≥16	32	≥64	≥64	≥64	≥64	≥64	≥32	≥32	≥128	≥32	≥16	≥64	≥4	≥8	≥320	1
7	R2	XDR	≥16	≥16	≥64	≥64	≥64	≥64	≥64	≥64	≥32	≥32	≥128	≥32	≥16	≥64	≥4	≥8	≥320	≤0.5
8	R3	XDR	≥16	≥16	32	≥64	≥64	≥64	≥64	≥64	≥32	≥32	≥128	≥32	≥16	≥64	≥4	≥8	≥320	2
9	R4	XDR	≥16	≥16	≥64	≥64	≥64	≥64	≥64	≥64	≥32	≥32	≥128	≥32	≥16	≥64	≥4	≥8	≥320	1
10	R5	XDR	≥16	≥16	32	≥64	≥64	≥64	≥64	≥64	≥32	≥32	≥128	≥32	≥16	≥64	≥4	≥8	≥320	1

^†^ CLSI resistant breakpoints are used. Abbreviations are used for all antibacterial agents as follows: β-lactams {IMI = imipenem (R > 8 mg/L); MEM = meropenem (R > 8 mg/L); FEP = cefepime (R > 4 mg/L); CXM= cefuroxime (R > 8 mg/L); CTX = cefotaxime (R > 2 mg/L); CAZ = ceftazidime (R > 4 mg/L); CRO = ceftriaxone= (R > 2 mg/L); FOX = cefoxitin (R > 8 mg/L); AMP = ampicillin (R > 8); AMC = amoxicillin-clavulanic acid (R > 8 mg/L); TZP = piperacillin-tazobactam (R > 16 mg/L)}; AMX = amoxicillin (R > 8 mg/L)}; Aminoglycosides {GEN = gentamicin (R > 4 mg/L); AMK = amikacin (R > 16 mg/L)}; Macrolide {ERT = erythromycin (R > 4)}; Fluoroquinolone {CIP = ciprofloxacin (R > 0.5 mg/L)}; Sulfonamides {SXT = trimethoprim-sulfamethoxazole (R > 4 mg/L)}; Glycylcyclines {TGC = tigecycline (R > 2 mg/L)}. * Categorized as MDR, XDR or PDR according to standard criteria [43].

**Table 2:** Relevant patient data, source of specimens, phenotypic, and genotypic characteristics of CRKP isolates.

Isolate *		Patient’s		Isolate’s		Carba NP ^†^	β-Lactamase Genes	In-Silico Typing		
No.	Strain ID	Sex	Age (Years)	Date	Source			MLST	*K Typing*	Allelic Types
1	B1	M ^‡^	24	15/04/2017	Blood	+	NDM-1, OXA-1, CTX-M-15, TEM-1B, SHV-1	ST152	KL149	*wzc*:928, *wzi*:110
2	B2	F ^§^	14	29/01/2017	Blood	+	NDM-1, ---------, CTX-M-15, TEM-1B, SHV-1	ST152	KL149	*wzc*:928, *wzi*:110
3	B3	M	30	03/01/2017	Blood	+	NDM-1, OXA-1, CTX-M-15, TEM-1B, SHV-1	ST152	KL149	*wzc*:928, *wzi*:110
4	B4	M	15 days	21/03/2017	Blood	+	NDM-1, OXA-1, CTX-M-15, TEM-1B, SHV-1	ST152	KL149	*wzc*:928, *wzi*:110
5	B5	-	8 months	25/04/2017	Blood	+	NDM-1, OXA-1, CTX-M-15, TEM-1B, SHV-1	ST152	KL149	*wzc*:928, *wzi*:110
6	R1	F	61	20/05/2016	Rectal	+	NDM-1, OXA-1, CTX-M-15, TEM-1B, SHV-1	ST3136	KL149	*wzc*:928, *wzi*:110
7	R2	F	72	18/07/2016	Rectal	+	NDM-1, OXA-1, CTX-M-15, TEM-1B, SHV-1	ST152	KL149	*wzc*:928, *wzi*:110
8	R3	M	25	13/06/2016	Rectal	+	NDM-1, OXA-1, CTX-M-15, TEM-1B, SHV-1	ST152	KL149	*wzc*:928, *wzi*:110
9	R4	F	21	11/07/2016	Rectal	+	NDM-1, OXA-1, CTX-M-15, TEM-1B, SHV-1	ST152	KL149	*wzc*:928, *wzi*:110
10	R5	F	66	27/07/2016	Rectal	+	NDM-1, OXA-1, CTX-M-15, TEM-1B, SHV-1	ST152	KL149	*wzc*:928, *wzi*:110

* Taxonomy determined by NCBI by comparing to proxytype strains in GenBank using the average nucleotide identity (ANI) test [44]. ^†^ Carba NP test for the detection of carbapenemase activity (+). ^‡^ Male. ^§^ Female.—Missing data. MLST—multilocus sequence typing, *K typing*—Klebsiella surface polysaccharide capsule characterization, *wzc* and *wzi* type—allelic typing scheme.

**Table 3:** Genetic environment of carbapenemase-encoding *bla*_NDM-1_ genetic structure borne on a plasmid with other mobile genetic elements.

Bacterial Strain		Carbapenemase ^1^	Plasmids Structure ^2^ (% Identity) ^3^	Plasmid Replicon Types	Plasmid MLST (Pmlsts)	Insertion Sequences	Intact Prophage
No.	ID						
1	B1	NDM-1:bleMBL	p18-43_01-like [100%]	IncFIB(K), IncFII, IncFIB(pB171), IncFII(Yp), ColRNAI	IncF[K12:A-:B36]	IS6, ISL3, IS256, IS3	10
2	B2	NDM-1:bleMBL	p18-43_01-like [99%]	IncFIB(K), IncFII, IncFIB(pB171), IncFII(Yp), ColRNAI	IncF[K12:A-:B36]	IS1182, IS5, ISNCY, ISL3	10
3	B3	NDM-1:bleMBL	p18-43_01-like [99%]	IncFIB(K), IncFII, IncFIB(pB171), IncFII(Yp), ColRNAI	IncF[K12:A-:B36]	IS6, ISL3, IS256, IS3	10
4	B4	NDM-1:bleMBL	p18-43_01-like [100%]	IncFIB(K), IncFII, IncFIB(pB171), IncFII(Yp), ColRNAI, Col440I	IncF[K12:A-:B36]	ISL3, IS256, IS481, IS21	10
5	B5	NDM-1:bleMBL	p18-43_01-like [100%]	IncFIB(K), IncFII, IncFIB(pB171), IncFII(Yp), ColRNAI, Col440I	IncF[K12:A-:B36]	IS6, IS66, IS1182, ISL3	10
6	R1	NDM-1:bleMBL	p18-43_01-like [100%]	IncFIB(K), IncFII, IncFIB(pB171),IncFII(Yp), ColRNAI	IncF[K12:A-:B36]	IS6, ISL3, IS256, IS3	10
7	R2	NDM-1:bleMBL	p18-43_01-like [99%]	IncFIB(K), IncFII, IncFIB(pB171), IncFII(Yp),ColRNAI	IncF[K12:A-:B36]	IS6, IS66, IS1182, ISL3	10
8	R3	NDM-1:bleMBL	p18-43_01-like [99%]	IncFIB(K), IncFII, IncFIB(pB171), IncFII(Yp), ColRNAI, Col440I	IncF[K12:A-:B36]	IS6, IS66, IS1182, ISL3	10
9	R4	NDM-1:bleMBL	p18-43_01-like [100%]	IncFIB(K), IncFII, IncFIB(pB171), IncFII(Yp), ColRNAI	IncF[K12:A-:B36]	IS1595, ISLre2, IS5, IS4	10
10	R5	NDM-1:bleMBL	p18-43_01-like [100%]	IncFIB(K), IncFII, IncFIB(pB171), IncFII(Yp), ColRNAI	IncF[K12:A-:B36]	IS6, ISL3, IS256, IS3	10

^1^ All the *bla*NDM-1 genes always occurred with bleomycin resistance determinants (bleMBL). ^2^ Referred to as “-like” when plasmid sequence is not circularized, but the carbapenemase-encoding contig revealed 99–100% nucleotide identity or synteny to the given plasmid. ^3^ Unless otherwise stated, all queries are of 100% coverage to subject/reference sequences.

## Supplementary Materials

The following are available online at https://www.mdpi.com/2076-2607/8/1/137/s1, Table S1: Genomic attributes of the 10 sequenced CRKP isolates. Table S2: Genomic analysis of other resistomes in Klebsiella pneumoniae (*n* = 10) from WGS data. Table S3: A table showing the diversity of MLST (ST types) and allelic profiles of the 7 housekeeping genes in the *XDR K. pneumoniae* isolates (*n* = 10). Figure S1: The total number of each predicted insertion sequence (IS) families via the ISFINDER database platform (https://isfinder.biotoul.fr/) in the isolates.

## References

[1] Roca I., Akova M., Baquero F., Carlet J., Cavaleri M., Coenen S., Cohen J., Findlay D., Gyssens I., Heure O.E. (2015). The global threat of antimicrobial resistance: Science for intervention. New Microbes New Infect..

[2] Prestinaci F., Pezzotti P., Pantosti A. (2015). Antimicrobial resistance: A global multifaceted phenomenon. Pathog. Glob. Health.

[3] Logan L.K., Weinstein R.A. (2017). The Epidemiology of Carbapenem-Resistant Enterobacteriaceae: The Impact and Evolution of a Global Menace. J. Infect. Dis..

[4] van Duin D., Doi Y. (2017). The global epidemiology of carbapenemase-producing Enterobacteriaceae. Virulence.

[5] Bonomo R.A., Burd E.M., Conly J., Limbago B.M., Poirel L., Segre J.A., Westblade L.F. (2018). Carbapenemase-Producing Organisms: A Global Scourge. Clin. Infect. Dis..

[6] Codjoe F., Donkor E. (2017). Carbapenem Resistance: A Review. Med. Sci..

[7] Carlet J., Jarlier V., Harbarth S., Voss A., Goossens H., Pittet D. (2012). Ready for a world without antibiotics? The Pensières Antibiotic Resistance Call to Action. Antimicrob. Resist. Infect. Control.

[8] Saeed A., Bosch A., Bettiol M., Nossa González D.L., Erben M.F., Lamberti Y. (2018). Novel guanidine compound against multidrug-resistant cystic fibrosis-associated bacterial species. Molecules.

[9] Patil M., Noonikara-Poyil A., Joshi S.D., Patil S.A., Patil S.A., Bugarin A. (2019). New Urea Derivatives as Potential Antimicrobial Agents: Synthesis, Biological Evaluation, and Molecular Docking Studies. Antibiotics.

[10] Somboro A.M., Amoako D.G., Osei Sekyere J., Kumalo H.M., Khan R., Bester L.A., Essack S.Y. (2019). 1,4,7-Triazacyclononane Restores the Activity of β-Lactam Antibiotics against Metallo-β-Lactamase-Producing Enterobacteriaceae: Exploration of Potential Metallo-β-Lactamase Inhibitors. Appl. Environ. Microbiol..

[11] Somboro A.M., Osei Sekyere J., Amoako D.G., Kumalo H.M., Khan R., Bester L.A., Essack S.Y. (2019). In vitro potentiation of carbapenems with tannic acid against carbapenemase-producing enterobacteriaceae: Exploring natural products as potential carbapenemase inhibitors. J. Appl. Microbiol..

[12] Papp-Wallace K.M., Endimiani A., Taracila M.A., Bonomo R.A. (2011). Carbapenems: Past, Present, and Future. Antimicrob. Agents Chemother..

[13] Kanj S.S., Kanafani Z.A. (2011). Current Concepts in Antimicrobial Therapy Against Resistant Gram-Negative Organisms: Extended-Spectrum β-Lactamase–Producing Enterobacteriaceae, Carbapenem-Resistant Enterobacteriaceae, and Multidrug-Resistant Pseudomonas aeruginosa. Mayo Clin. Proc..

[14] Queenan A.M., Bush K. (2007). Carbapenemases: The versatile β-lactamases. Clin. Microbiol. Rev..

[15] Cui X., Zhang H., Du H. (2019). Carbapenemases in Enterobacteriaceae: Detection and Antimicrobial Therapy. Front. Microbiol..

[16] Pitout J.D.D., Nordmann P., Poirel L. (2015). Carbapenemase-Producing Klebsiella pneumoniae, a Key Pathogen Set for Global Nosocomial Dominance. Antimicrob. Agents Chemother..

[17] Schwaber M.J., Carmeli Y. (2008). Carbapenem-resistant enterobacteriaceae: A potential threat. JAMA.

[18] Fasciana T., Gentile B., Aquilina M., Ciammaruconi A., Mascarella C., Anselmo A., Fortunato A., Fillo S., Petralito G., Lista F. (2019). Co-existence of virulence factors and antibiotic resistance in new Klebsiella pneumoniae clones emerging in south of Italy. BMC Infect. Dis..

[19] Kieffer N., Nordmann P., Aires-de-Sousa M., Poirel L. (2016). High Prevalence of Carbapenemase-Producing Enterobacteriaceae among Hospitalized Children in Luanda, Angola. Antimicrob. Agents Chemother..

[20] Sangare S.A., Rondinaud E., Maataoui N., Maiga A.I., Guindo I., Maiga A., Camara N., Dicko O.A., Dao S., Diallo S. (2017). Very high prevalence of extended-spectrum beta-lactamase-producing Enterobacteriaceae in bacteriemic patients hospitalized in teaching hospitals in Bamako, Mali. PLoS ONE.

[21] Abderrahim A., Djahmi N., Pujol C., Nedjai S., Bentakouk M.C., Kirane-Gacemi D., Dekhil M., Sotto A., Lavigne J.-P., Pantel A. (2017). First Case of NDM-1-Producing Klebsiella pneumoniae in Annaba University Hospital, Algeria. Microb. Drug Resist..

[22] Moussounda M., Diene S.M., Dos Santos S., Goudeau A., François P., van der Mee-Marquet N. (2017). Emergence of blaNDM-7–producing enterobacteriaceaein Gabon, 2016. Emerg. Infect. Dis..

[23] Jesumirhewe C., Springer B., Lepuschitz S., Allerberger F., Ruppitsch W. (2017). Carbapenemase-Producing Enterobacteriaceae Isolates from Edo State, Nigeria. Antimicrob. Agents Chemother..

[24] Rubin J.E., Peirano G., Peer A.K., Govind C.N., Pitout J.D.D. (2014). NDM-1–producing Enterobacteriaceae from South Africa: Moving towards endemicity?. Diagn. Microbiol. Infect. Dis..

[25] Carattoli A. (2009). Resistance Plasmid Families in Enterobacteriaceae. Antimicrob. Agents Chemother..

[26] Poirel L., Dortet L., Bernabeu S., Nordmann P. (2011). Genetic Features of bla NDM-1 -Positive Enterobacteriaceae. Antimicrob. Agents Chemother..

[27] Potter R.F., D’Souza A.W., Dantas G. (2016). The rapid spread of carbapenem-resistant Enterobacteriaceae. Drug Resist. Updat..

[28] Li L., Yu T., Ma Y., Yang Z., Wang W., Song X., Shen Y., Guo T., Kong J., Wang M. (2019). The genetic structures of an extensively drug resistant (XDR) Klebsiella pneumoniae and its plasmids. Front. Cell. Infect. Microbiol..

[29] Gijon D., Curiao T., Baquero F., Coque T.M., Canton R. (2012). Fecal Carriage of Carbapenemase-Producing Enterobacteriaceae: A Hidden Reservoir in Hospitalized and Nonhospitalized Patients. J. Clin. Microbiol..

[30] Pournaras S., Zarkotou O., Poulou A., Kristo I., Vrioni G., Themeli-Digalaki K., Tsakris A. (2013). A Combined Disk Test for Direct Differentiation of Carbapenemase-Producing Enterobacteriaceae in Surveillance Rectal Swabs. J. Clin. Microbiol..

[31] Perry J.D. (2017). A Decade of Development of Chromogenic Culture Media for Clinical Microbiology in an Era of Molecular Diagnostics. Clin. Microbiol. Rev..

[32] Clinical and Laboratory Standards Institute (2017). Performance Standards for Antimicrobial Susceptibility Testing: 27th Edition Informational Supplement M100-S27.

[33] Vasoo S. (2017). Susceptibility Testing for the Polymyxins: Two Steps Back, Three Steps Forward?. J. Clin. Microbiol..

[34] Bankevich A., Nurk S., Antipov D., Gurevich A.A., Dvorkin M., Kulikov A.S., Lesin V.M., Nikolenko S.I., Pham S., Prjibelski A.D. (2012). SPAdes: A new genome assembly algorithm and its applications to single-cell sequencing. J. Comput. Biol..

[35] Aziz R.K., Bartels D., Best A., DeJongh M., Disz T., Edwards R.A., Formsma K., Gerdes S., Glass E.M., Kubal M. (2008). The RAST Server: Rapid annotations using subsystems technology. BMC Genom..

[36] Zankari E., Hasman H., Cosentino S., Vestergaard M., Rasmussen S., Lund O., Aarestrup F.M., Larsen M.V. (2012). Identification of acquired antimicrobial resistance genes. J. Antimicrob. Chemother..

[37] Jia B., Raphenya A.R., Alcock B., Waglechner N., Guo P., Tsang K.K., Lago B.A., Dave B.M., Pereira S., Sharma A.N. (2017). CARD 2017: Expansion and model-centric curation of the comprehensive antibiotic resistance database. Nucleic Acids Res..

[38] Carattoli A., Zankari E., García-Fernández A., Voldby Larsen M., Lund O., Villa L., Møller Aarestrup F., Hasman H. (2014). In Silico Detection and Typing of Plasmids using PlasmidFinder and Plasmid Multilocus Sequence Typing. Antimicrob. Agents Chemother..

[39] Zhou Y., Liang Y., Lynch K.H., Dennis J.J., Wishart D.S. (2011). PHAST: A Fast Phage Search Tool. Nucleic Acids Res..

[40] Siguier P. (2006). ISfinder: The reference centre for bacterial insertion sequences. Nucleic Acids Res..

[41] Ahrenfeldt J., Skaarup C., Hasman H., Pedersen A.G., Aarestrup F.M., Lund O. (2017). Bacterial whole genome-based phylogeny: Construction of a new benchmarking dataset and assessment of some existing methods. BMC Genom..

[42] Hadfield J., Croucher N.J., Goater R.J., Abudahab K., Aanensen D.M., Harris S.R. (2017). Phandango: An interactive viewer for bacterial population genomics. Bioinformatics.

[43] Magiorakos A., Srinivasan A., Carey R.B., Carmeli Y., Falagas M.E., Giske C.G., Harbarth S., Hindler J.F. (2011). bacteria: An international expert proposal for interim standard definitions for acquired resistance. Clin. Microbiol. Infect..

[44] Jain C., Rodriguez-R L.M., Phillippy A.M., Konstantinidis K.T., Aluru S. (2018). High throughput ANI analysis of 90K prokaryotic genomes reveals clear species boundaries. Nat. Commun..

[45] Ramsamy Y., Mlisana K.P., Allam M., Ismail A., Singh R., Amoako D.G., Essack S.Y. (2018). Whole-Genome Sequence of a Novel Sequence Type 3136 Carbapenem-Resistant Klebsiella pneumoniae Strain Isolated from a Hospitalized Patient in Durban, South Africa. Microbiol. Resour. Announc..

[46] Sugawara Y., Hagiya H., Akeda Y., Aye M.M., Myo Win H.P., Sakamoto N., Shanmugakani R.K., Takeuchi D., Nishi I., Ueda A. (2019). Dissemination of carbapenemase-producing Enterobacteriaceae harbouring blaNDM or blaIMI in local market foods of Yangon, Myanmar. Sci. Rep..

[47] Sheu C.-C., Chang Y.-T., Lin S.-Y., Chen Y.-H., Hsueh P.-R. (2019). Infections Caused by Carbapenem-Resistant Enterobacteriaceae: An Update on Therapeutic Options. Front. Microbiol..

[48] Lee C.-R., Lee J.H., Park K.S., Kim Y.B., Jeong B.C., Lee S.H. (2016). Global Dissemination of Carbapenemase-Producing Klebsiella pneumoniae: Epidemiology, Genetic Context, Treatment Options, and Detection Methods. Front. Microbiol..

[49] Naparstek L., Carmeli Y., Chmelnitsky I., Banin E., Navon-Venezia S. (2012). Reduced susceptibility to chlorhexidine among extremely-drug-resistant strains of Klebsiella pneumoniae. J. Hosp. Infect..

[50] Yang J., Ye L., Wang W., Luo Y., Zhang Y., Han L. (2011). Diverse prevalence of 16S rRNA methylase genes armA and rmtB amongst clinical multidrug-resistant Escherichia coli and Klebsiella pneumoniae isolates. Int. J. Antimicrob. Agents.

[51] Malande O.O., Du Plessis A., Rip D., Bamford C., Eley B. (2016). Invasive carbapenem-resistant Enterobacteriaceae infection at a paediatric hospital: A case series. S. Afr. Med. J..

[52] Kang J.S., Yi J., Ko M.K., Lee S.O., Lee J.E., Kim K.-H. (2019). Prevalence and Risk Factors of Carbapenem-resistant Enterobacteriaceae Acquisition in an Emergency Intensive Care Unit in a Tertiary Hospital in Korea: A Case-Control Study. J. Korean Med. Sci..

[53] Li G., Zhao S., Wang S., Sun Y., Zhou Y., Pan X. (2019). A 7-year surveillance of the drug resistance in Klebsiella pneumoniae from a primary health care center. Ann. Clin. Microbiol. Antimicrob..

[54] Salomão M.C., Guimarães T., Duailibi D.F., Perondi M.B.M., Letaif L.S.H., Montal A.C., Rossi F., Cury A.P., Duarte A.J.S., Levin A.S. (2017). Carbapenem-resistant Enterobacteriaceae in patients admitted to the emergency department: Prevalence, risk factors, and acquisition rate. J. Hosp. Infect..

[55] Nicolau D., Zmarlicka M., Nailor M. (2015). Impact of the New Delhi metallo-beta-lactamase on beta-lactam antibiotics. Infect. Drug Resist..

[56] Somboro A.M., Osei Sekyere J., Amoako D.G., Essack S.Y., Bester L.A. (2018). Diversity and Proliferation of Metallo-β-Lactamases: A Clarion Call for Clinically Effective Metallo-β-Lactamase Inhibitors. Appl. Environ. Microbiol..

[57] Lowman W., Sriruttan C., Nana T., Bosman N., Duse A., Venturas J., Clay C., Coetzee J. (2011). NDM-1 has arrived: First report of a carbapenem resistance mechanism in South Africa. S. Afr. Med. J..

[58] Pedersen T., Sekyere J.O., Govinden U., Moodley K., Sivertsen A., Samuelsen Ø., Essack S.Y., Sundsfjord A. (2018). Spread of Plasmid-Encoded NDM-1 and GES-5 Carbapenemases among Extensively Drug-Resistant and Pandrug-Resistant Clinical Enterobacteriaceae in Durban, South Africa. Antimicrob. Agents Chemother..

[59] Osei Sekyere J. (2016). Current State of Resistance to Antibiotics of Last-Resort in South Africa: A Review From a Public Health Perspective. Front. Public Health.

[60] Sekyere J.O., Govinden U., Essack S. (2015). The Molecular Epidemiology and Genetic Environment of Carbapenemases Detected in Africa. Microb. Drug Resist..

[61] Khan A.U., Maryam L., Zarrilli R. (2017). Structure, Genetics and Worldwide Spread of New Delhi Metallo-β-lactamase (NDM): A threat to public health. BMC Microbiol..

[62] Osei Sekyere J., Amoako D.G. (2017). Carbonyl Cyanide m-Chlorophenylhydrazine (CCCP) Reverses Resistance to Colistin, but Not to Carbapenems and Tigecycline in Multidrug-Resistant Enterobacteriaceae. Front. Microbiol..

[63] Agyepong N., Govinden U., Owusu-Ofori A., Amoako D.G., Allam M., Janice J., Pedersen T., Sundsfjord A., Essack S. (2019). Genomic characterization of multidrug-resistant ESBL-producing Klebsiella pneumoniae isolated from a Ghanaian teaching hospital. Int. J. Infect. Dis..

[64] Osei Sekyere J., Amoako D.G. (2017). Genomic and phenotypic characterisation of fluoroquinolone resistance mechanisms in Enterobacteriaceae in Durban, South Africa. PLoS ONE.

[65] Wyres K.L., Wick R.R., Gorrie C., Jenney A., Follador R., Thomson N.R., Holt K.E. (2016). Identification of Klebsiella capsule synthesis loci from whole genome data. Microb. Genom..

[66] Wyres K.L., Nguyen T.N., Lam M.M., Judd L.M., van Vinh Chau N., Dance D.A., Ip M., Karkey A., Ling C.L., Miliya T. (2019). Genomic surveillance for hypervirulence and multi-drug resistance in invasive Klebsiella pneumoniae from south and southeast Asia. bioRxiv.

[67] Bi W., Liu H., Dunstan R.A., Li B., Torres V.V.L., Cao J., Chen L., Wilksch J.J., Strugnell R.A., Lithgow T. (2017). Extensively drug-resistant klebsiella pneumoniae causing nosocomial bloodstream infections in China: Molecular investigation of antibiotic resistance determinants, Informing therapy, and clinical outcomes. Front. Microbiol..

[68] Carattoli A., Seiffert S.N., Schwendener S., Perreten V., Endimiani A. (2015). Differentiation of IncL and IncM Plasmids Associated with the Spread of Clinically Relevant Antimicrobial Resistance. PLoS ONE.

[69] Kocsis E., Gužvinec M., Butić I., Krešić S., Crnek S.Š., Tambić A., Cornaglia G., Mazzariol A. (2016). bla NDM-1 Carriage on IncR Plasmid in Enterobacteriaceae Strains. Microb. Drug Resist..

[70] Sahl J.W., Johnson J.K., Harris A.D., Phillippy A.M., Hsiao W.W., Thom K.A., Rasko D.A. (2011). Genomic comparison of multi-drug resistant invasive and colonizing Acinetobacter baumannii isolated from diverse human body sites reveals genomic plasticity. BMC Genom..

[71] Amoako D.G., Somboro A.M., Abia A.L.K., Allam M., Ismail A., Bester L., Essack S.Y. (2019). Genomic analysis of methicillin-resistant Staphylococcus aureus isolated from poultry and occupational farm workers in Umgungundlovu District, South Africa. Sci. Total Environ..

[72] Polz M.F., Alm E.J., Hanage W.P. (2013). Horizontal gene transfer and the evolution of bacterial and archaeal population structure. Trends Genet..

[73] Daubin V., Szöllősi G.J. (2016). Horizontal Gene Transfer and the History of Life. Cold Spring Harb. Perspect. Biol..

[74] Mantere T., Kersten S., Hoischen A. (2019). Long-Read Sequencing Emerging in Medical Genetics. Front. Genet..

[75] Russotto V., Cortegiani A., Fasciana T., Iozzo P., Raineri S.M., Gregoretti C., Giammanco A., Giarratano A. (2017). What Healthcare Workers Should Know about Environmental Bacterial Contamination in the Intensive Care Unit. Biomed. Res. Int..

[76] Magiorakos A.P., Burns K., Rodríguez Baño J., Borg M., Daikos G., Dumpis U., Lucet J.C., Moro M.L., Tacconelli E., Simonsen G.S. (2017). Infection prevention and control measures and tools for the prevention of entry of carbapenem-resistant Enterobacteriaceae into healthcare settings: Guidance from the European Centre for Disease Prevention and Control. Antimicrob. Resist. Infect. Control.

