# Supplementary material for: Genomic Analysis of Carbapenemase-Producing Extensively Drug-Resistant Klebsiella pneumoniae Isolates Reveals the Horizontal Spread of p18-43_01 Plasmid Encoding blaNDM-1 in South Africa
Source: Microorganisms. 2020 Jan 17;8(1):137. doi: 10.3390/microorganisms8010137 (PMC7023316; doi:10.3390/microorganisms8010137)
Supplement: Supplementary file 1 [file microorganisms-08-00137-s001.pdf]

Table S1: Genomic attributes of the 10 sequenced CRKP isolates.

Isolate	Species	Accession Number	Size (Mbp)	GC Content	No. of contigs	N50	L50	RNAs	tRNAs	Protein coding sequences	Average Coverage (X)
B1	*K. pneumoniae*	QMBR00000000	5.59	57.3	195	104657	14	112	82	5469	100.14
B2	*K. pneumoniae*	QMBU00000000	5.56	57.3	207	101862	15	110	81	5437	100.40
B3	*K. pneumoniae*	QMBV00000000	5.70	57.3	309	101223	18	115	82	5651	98.12
B4	*K. pneumoniae*	QMBT00000000	5.50	57.0	1837	5402	289	117	83	6597	102.17
B5	*K. pneumoniae*	QMBS00000000	5.64	57.3	263	107119	15	110	81	5577	99.08
R1	*K. pneumoniae*	QMCA00000000	5.57	57.4	180	103476	16	109	82	5427	100.33
R2	*K. pneumoniae*	QMBX00000000	5.59	57.4	147	175929	12	109	81	5447	100.30
R3	*K. pneumoniae*	QMBW00000000	5.59	57.3	150	184651	12	110	81	5459	100.12
R4	*K. pneumoniae*	QMBY00000000	5.59	57.4	181	152812	12	114	85	5454	100.06
R5	*K. pneumoniae*	QMBZ00000000	5.58	57.3	122	159521	12	113	84	5439	100.17

TABLE S2: Genomic analysis of other resistome in Klebsiella pneumoniae (n=10) from WGS data.

No.	Strain ID	Antibiotic classes/ resistance genes						
		Acquired resistance genes					Chromosomal mutation	
		Aminoglycosides	Phenicols	Trimethoprim	Sulphonamides	Fluoroquinolones	*GyrA*	*ParC*
1	B1	aac(3)-IIa, aadA16, rmtC, …………., strA, strB, aac(6')Ib-cr	catB4, catA1	dfrA27	sul1, sul2	oqxB, oqxA, aac(6')-Ib-cr, ……..	S83F, D87A	S80I
2	B2	aac(3)-IIa, ………., rmtC, …………, strA, strB, aac(6')Ib-cr	catB4, catA1	---------	sul1, sul2	oqxB, oqxA, aac(6')-Ib-cr, …….	S83F, D87A	S80I
3	B3	aac(3)-IIa, aadA16, rmtC, aph(3)-1a, strA, strB ……………	catB4, catA1	dfrA27	sul1, sul2	oqxB, oqxA, ……………, ……..	S83F, D87A	S80I
4	B4	aac(3)-IIa, aadA16, rmtC, aph(3)-1a, strA, strB, aac(6')Ib-cr	catB4, catA1	dfrA27	sul1, sul2	oqxB, oqxA, aac(6')-Ib-cr, QnrB6	S83F, D87A	S80I
5	B5	aac(3)-IIa, aadA16, rmtC, aph(3)-1a, strA, strB, aac(6')Ib-cr	catB4, catA1	dfrA27	sul1, sul2	oqxB, oqxA, aac(6')-Ib-cr, QnrB6	S83F, D87A	S80I
6	R1	aac(3)-IIa, aadA16, rmtC, aph(3)-1a, strA, strB, aac(6')Ib-cr	catB4, catA1	dfrA27	sul1, sul2	oqxB, oqxA, aac(6')-Ib-cr, QnrB6	S83F, D87A	S80I
7	R2	aac(3)-IIa, aadA16, rmtC, aph(3)-1a, strA, strB, aac(6')Ib-cr	catB4, catA1	dfrA27	sul1, sul2	oqxB, oqxA, aac(6')-Ib-cr, ………	S83F, D87A	S80I
8	R3	aac(3)-IIa, aadA16, rmtC, …………, strA, strB, aac(6')Ib-cr	catB4, catA1	dfrA27	sul1, sul2	oqxB, oqxA, aac(6')-Ib-cr, QnrB6	S83F, D87A	S80I
9	R4	aac(3)-IIa, aadA16, rmtC, ………...., strA, strB, aac(6')Ib-cr	catB4, catA1	dfrA27	sul1, sul2	oqxB, oqxA, aac(6')-Ib-cr, QnrB6	S83F, D87A	S80I
10	R5	aac(3)-IIa, aadA16, rmtC, ………...., strA, strB, aac(6')Ib-cr	catB4, catA1	dfrA27	sul1, sul2	oqxB, oqxA, aac(6')-Ib-cr, QnrB6	S83F, D87A	S80I

Unless otherwise stated in the footnote, *K. pneumoniae* ATCC 13883 (**PRJNA244567**) was used as a reference strain in the comparative genomic analysis to elucidate the chromosomal mutation in fluoroquinolone.

TABLE S3: A table showing the diversity of MLST (ST types) and allelic profiles of the 7 housekeeping genes in the *XDR K. pneumoniae* isolates (n = 10).

Number of isolates	MLST	*gapE*	*infB*	*mdh*	*pgi*	*phoE*	*rpoB*	*tnoB*
9	152	2	3	2	1	1	4	56
1	3136	2	3	2	1	1	85	56

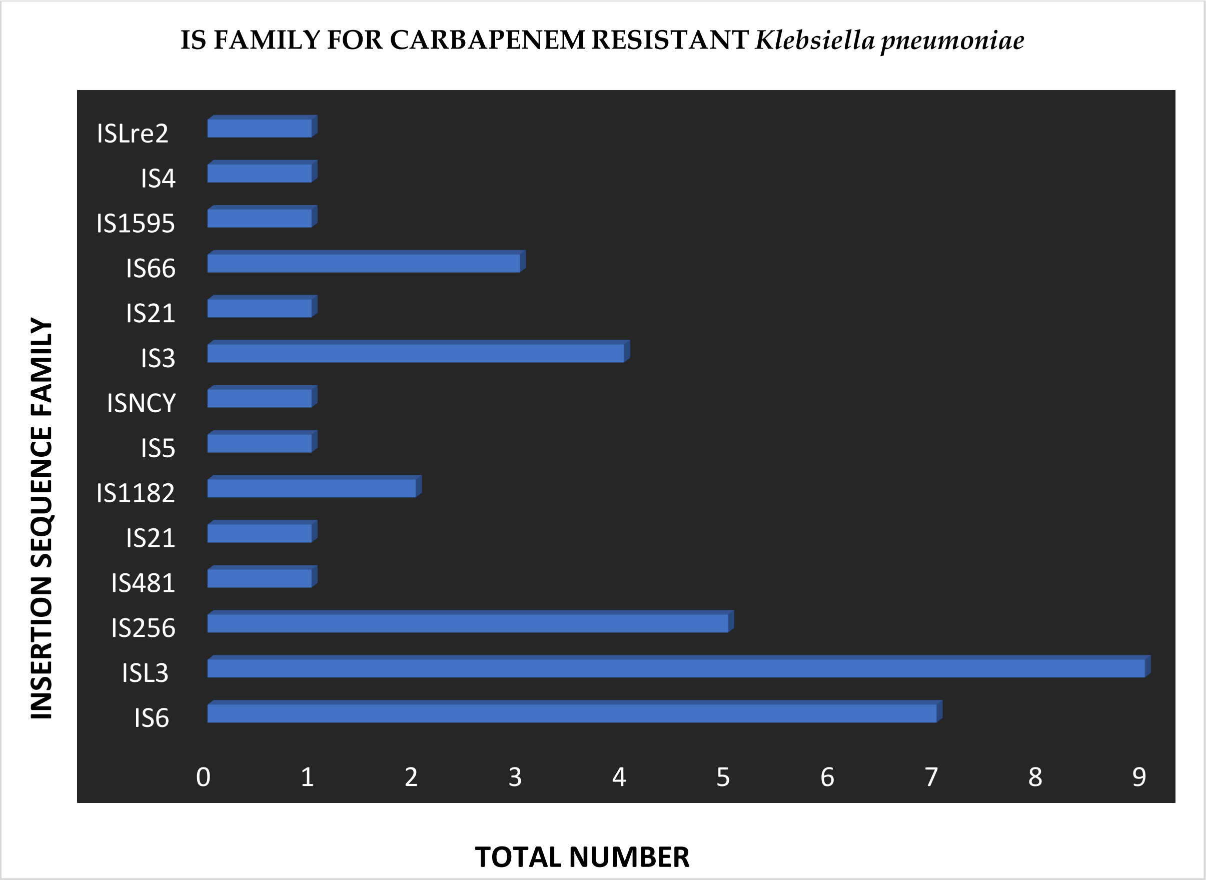

Figure S1: The total number of each predicted insertion sequence (IS) families via the ISFINDER database platform (https://isfinder.biotoul.fr/) in the isolates.
